# The Purdue Extension and Indiana CTSI’s Community Health Partnerships collaboration: An innovative, generalizable, state-wide model to help communities build a culture of health

**DOI:** 10.1017/cts.2017.300

**Published:** 2017-12-26

**Authors:** Dennis A. Savaiano, Krystal H. Lynch, Donna J. Vandergraff, Sarah E. Wiehe, Lisa K. Staten, Douglas K. Miller

**Affiliations:** 1 Department of Nutrition Science, Purdue University, West Lafayette, IN, USA; 2 Indiana Children’s Health Service Research, Department of Pediatrics, Indiana University School of Medicine, Indianapolis, IN, USA; 3 Indiana University Fairbanks School of Public Health, Indianapolis, IN, USA; 4 IU Center for Aging Research, Regenstrief Institute, Inc., Indianapolis, IN, USA

**Keywords:** Engaging Community Health Coalitions to improve health, dissemination and implementation, PSE interventions, interdisciplinary collaborative translational research, expanded funding agency collaboration

## Abstract

The Indiana Clinical and Translational Sciences Institute’s Community Engagement Partners-Purdue Extension collaborative model demonstrates tremendous potential for creating state-wide programmatic efforts and improvements in both the health culture and status of Indiana residents across the state. It can serve as a prototype not only for others interested in pursuing wide geographic health improvements through Clinical and Translational Sciences Award-Cooperative Extension partnerships but also for broader collaborations among United States Department of Agriculture, National Institutes of Health, Centers for Disease Control and Prevention, state and local health departments, and health foundation efforts to improve population health.

## An Archetypal Problem and a Potential Systems-Approach Solution

For years, Indiana has been one of the unhealthiest states in the nation. In 2016, Indiana ranked as the 39th out of 50 states for overall health. The state ranks in the bottom quartile of states for the following items: public health funding (2nd worst), air pollution (4th worst), infant mortality (8th worst), cancer death (9th worst), physical inactivity (11th worst), smoking rates (12th worst), and premature death (12th worst), and in the bottom third for the following: poor physical health (13th worst), diagnosed diabetes (14th worst), poor mental health days (14th worst), obesity (15th worst), and drug deaths (16th worst) [1]. Most Indiana counties are rural, economically disadvantaged, and relatively isolated with worse health status and risk factors than non-rural Indiana counties [1–4]. Indiana’s health challenges will be difficult to reverse, and together they can be characterized as a “wicked problem.” “Wicked” in this context does not signify that the problem is ethically appalling, but rather that the problem is “illusive or difficult to pin down and influenced by a constellation of complex social and political factors, some of which change during the process of solving the problem” and embedded in the fabric of communities. For these reasons, wicked problems are notoriously difficult to solve [5, 6].

Purdue University Extension has a long history of effective community engagement and programming throughout Indiana [7], and the more-recently established Indiana Clinical and Translational Sciences Institute (CTSI) Community Health Partnerships (CHeP) program has extensive programmatic experience and presence throughout Indiana (https://www.indianactsi.org/chep) as well. We believe the collaboration between Purdue Extension and CTSI-CHeP described in this report has the skills and experience; reach; effective programmatic approaches; and ability to identify, implement, and expand health promoting initiatives throughout the state; it also has the capacity to establish long-term organizational systems to sustain effective public health-focused programming [8]. Further, these ongoing systems and sustained programming will be well-positioned to (a) successfully address the wicked problem of poor health in Indiana and (b) demonstrate a collaborative model with all the required features [9] of an effective prototype that other regions of the nation can consider employing.

Specifically, Purdue Extension and CHeP are designing community-based collaborative models to expand health-related training, research, and programs throughout Indiana to advance health and health culture throughout the state. Since 2013 work has focused on advancing community health coalitions (CHCs) in several counties; understanding the factors that enhance those coalitions’ ability to improve the health of their target population; educating county Extension educators regarding how to employ policy, systems, and environmental (PSE) interventions; building partnerships between CTSI and county Extension faculty and practitioners; and constructing meaningful, active, long-term engagement of diverse stakeholders (which has recently been titled, “broadly engaged team science” [10]) as well as shared governance among the stakeholders. Future work will expand the effort to include additional counties, health conditions, CHCs, and partners in research and programming. This work has and will continue to focus on developing, enhancing, and sustaining an interactive network of learning and capacity-building communities, both within each coalition and across coalitions [11–13]. This state-wide model of CHeP and Extension collaboration should be transferable to other locales throughout the United States with significant potential to link (a) the long-standing Extension-community collaborative relationships with (b) CTSI-sponsored research, health-promotion programs, and implementation-science expertise and to unite these 2, in turn, with (c) additional important partners such as schools of public health and health departments. The background for this agenda and the systems approach to achieving it follow.

### Purdue Extension

*Purdue Extension* provides direct services to about 1.4 million Indiana residents (of 6.5 million living in the state; 22%) each year through its educational programs. Over the past 20 years, health and wellness promotion has become one of the fastest-growing areas of work for Purdue Extension. Extension educators, specialists, and volunteers live and work in all 92 Indiana counties and bi-directionally link Purdue’s research, programmatic, and educational capabilities with local initiatives, needs and partners (in collaborations that are often long-standing), frequently in collaboration with local and state health departments. Extension’s work developing local CHCs across the state has been and remains an instrumental partner in the CHeP program, linking implementation scientists and other researchers from the broader scientific community of CTSI with local partners to develop flourishing health coalitions, learning communities, research partnerships, and capacity-building alliances [14].

### CTSI-CHeP

The Indiana CTSI has state-wide reach via its 4 constituent campuses (Indiana University, Bloomington; Indiana University, Indianapolis; Purdue University; and Notre Dame) and the numerous external collaborations emanating from those 4 campuses. Indiana University is the only medical school in the state, and the university’s 2 schools of public health (in Indianapolis and Bloomington) constitute the only schools of public health in Indiana; all 3 are active participants in the Indiana CTSI. Since 2008, CHeP has generated productive activities, robust networks, and early-stage outcomes that are advancing community-engaged research and programming on all 4 Indiana CTSI campuses and throughout the state via partnerships with many community and state-level entities (e.g., Indiana State Department of Health, Indiana Minority Health Coalition, and Indiana Rural Health Association). The great majority of this work focuses on PSE interventions as the avenue to creating a healthier lifestyle and better health outcomes across Indiana.

This interacting network of components and partnerships is effectively coordinated via newsletters, an active Web site (https://www.indianactsi.org/chep), pilot program funding and pilot awardee meetings, liaisons from dispersed community and academic partners to the central CHeP Leadership group, etc. Notably, CHeP’s multilayered collaborative network of engaged entities now includes more than 650 member partners. CHeP has funded 43 community-engaged projects that involve at least 1 community partner and 1 university partner and use state-of-the-art principles of community-based participatory research [11] and of broadly engaged team science [10]. As a result of this program, the number of proposals for extramural funding from CHeP-supported projects has increased from 1 in 2009 to 15–28 per year in 2013–2016. CHeP/Extension partnerships have also developed at the state level with the Indiana State Department of Health (which contributes seed dollars for CHeP’s annual pilot grant awards) and collaboration on health coalition development through the Indiana Public Health Association Healthy Weight Initiative (http://www.indianaobesity.org/).

### Recent Progress

These collaborative efforts may be beginning to bear fruit. From 2015 to 2016, Indiana experienced improvements in some health indicators (especially smoking, obesity, and overall health) [1]. CHeP-Extension cannot take all the credit for these improvements, but very likely it did contribute to this success.

## National Resources Available for Assisting Community-Based Interventions to Improve Health

### The Clinical and Translational Science Award (CTSA) Program

With support of National Institutes of Health (NIH), the CTSA program was launched in 2006; funded awardee institutions are tasked with working with multiple partners to accelerate progress toward improved health across the country (https://ctsacentral.org/). This program currently involves 62 institutions with CTSAs distributed throughout the 50 states and District of Columbia (DC). Of these, 20 states have 1 CTSA; DC and 13 states have 2 or more CTSAs; and only 18 states lack any CTSA [15]. Essentially all CTSAs have 1 or more components involved in community-engaged work, although the extent and vigor of that work vary significantly across the CTSAs, as does the robustness of relationships with schools of public health and state/local health departments. Community engagement activities across CTSAs are coordinated by 2 primary mechanisms: (a) the Collaboration/Engagement Task Force is comprised of representatives from all of the CTSAs and from NIH (https://ctsacentral.org/articles/?article=Collaboration%20Engagement) and (b) the Partners for the Advancement of Community Engaged Research (PACER) Special Interest Group of the CTSA (http://www.actscience.org/page/SIGS), augmented by smaller collaborations among member institutions. Thus, a robust infrastructure is in place to not only support Extension-CTSA community engagement collaborations within the 33 states and DC but to also coordinate across CTSA site-specific alliances.

### Cooperative Extension

Health-focused extension resources exist in all states with significant potential for collaboration with local CTSAs and related partners. Extension-allied, health-related initiatives have a long history in this country and continue to provide a robust infrastructure for improving health throughout every state. The land-grant system has a very long history of improving health by enhancing the quality of food supply [16]. Since World War II, Extension has promoted access to health care and provided community-based education in nutrition, food safety and preparation, and environmental safety. With the formation of the United States Drug Administration’s (USDA’s) National Institute of Food and Agriculture (NIFA) in 2008, USDA’s role in promoting health continued to grow. Currently NIFA’S and Extension’s health promotional efforts include educational and intervention programs in (1) nutrition, (2) obesity and healthy weight, (3) hunger and food security, (4) health and wellness, (5) food safety and biosecurity, (6) food science and technology, and (7) environmental health (https://nifa.usda.gov/topics). Extension Services also have responsibility for the Federal Expanded Food and Nutrition Education Program and often for Supplemental Nutrition Assistance Program (SNAP)-Education programs.

The well-known 4H program has always had health as one of its 4 personal development areas: head, heart, hands, and health. The 4H curriculum, which emphasizes experiential “Learn by Doing” includes modules on Healthy Living and STEM/Science [17], and both of these modules could easily be expanded substantially. The potential for 4H to amplify efforts around healthy living and science, collaborating with the medical community outreach through CTSA engagement mechanisms could stimulate a dramatic cultural shift to make health a priority for youth development, first in rural areas and subsequently in urban ones.

Community development and capacity building in communities with the most needy and unhealthy members of our society have long been priorities for Extension [18]. Recent focus throughout Extensions’ multiple programs on capacity building for health programming plus PSE interventions and research has positioned Extension with unique experiences and opportunities in both rural and urban areas. Notably, these foci coincide nicely with NIH and CDC efforts to increase local capacities and promote PSE interventions.

### Potential to Generalize the Indiana CHeP-Extension Collaborative Model to Other Regions and States

Extension’s robust presence in all states and its rich national programs and resources plus CTSAs’ active involvement in advancing health in 33 states and DC creates a dynamic infrastructure for CTSA-Extension program development, implementation, dissemination, and assessment throughout much of the country. However, this potential is severely underutilized and would be greatly advanced by better mechanisms for funding this type of collaborative effort.

## Collaboration on Steroids: The Potent Synergistic Potential for Combining USDA-DHHS Programming and Funding

The CHeP-Extension model (and its logical duplication in other states) is a compelling example of what can be accomplished with collaboration between USDA-funded and NIH-funded programs and demonstrates the tremendous potential for collaboration among national level organizational programmatic efforts and funding. Specifically, with (a) the relatively recent convergence of goals and approaches regarding improving population health in the USDA, NIH, and CDC and (b) the synergistic expertise contained in these organizations, collaboration across agencies could provide immense stimulus to improving population health. However, to date collaborations among all 3 have been nearly nonexistent and collaborations between any 2 have been infrequent. USDA and NIH collaborate on developing the Dietary Guidelines for Americans to promote nutrient dense healthy diets, and NIH and CDC collaborate on supporting the National Health and Nutrition Examination Survey (NHANES). The National Collaborative on Childhood Obesity Research is a joint effort of NIH, CDC, USDA, and the Robert Wood Johnson Foundation [19]. While these collaborations are useful, relatively few resources have been contributed to multiagency efforts that would promote health and prevent disease. However, the time may be ripe to work toward greater collaboration and interactive cooperation, particularly in funding joint agency efforts.

We argue that neither NIH, USDA, nor CDC alone has sufficient resources invested in translational research efforts to improve health at the local level with any reach, rapidity, or scalability. But collectively and in partnership with others (e.g., universities, the Robert Wood Johnson and Kellogg foundations, and state and local health departments), much could be accomplished. The potential to develop effective collaborations, approaches, and resources to address the wicked problem of health is so great that we must not let historical boundaries of narrow purpose, unique political supporters, and existing infrastructures limit our creativity to work together to improve the health of all Americans.

## Conclusions

The CHeP-Purdue Extension collaborative model demonstrates great potential for creating state-wide improvements in both health culture and health status. It can serve as a prototype not only for others interested in pursuing wide geographic health improvements through CTSA-Extension partnerships but also for broader collaborations among USDA, NIH, CDC, state and local health departments, and health foundation efforts to improve population health. The expansion of multiagency, multilevel collaborations could greatly advance the reach, speed, success, and sustainability of health-improving efforts throughout the country and together solve the wicked population health challenges that this country faces.
